# Cross-Disciplinary Collaboration to Promote Trauma-Informed Practices in Early Childhood and Primary Education

**DOI:** 10.1177/15248380251325217

**Published:** 2025-03-27

**Authors:** Yihan Sun, Helen Skouteris, Andrea Tamblyn, Emily Berger, Claire Blewitt

**Affiliations:** 1 Monash University, Melbourne, VIC, Australia

**Keywords:** trauma-informed, early childhood, primary school, cross-disciplinary collaboration, cross-sector collaboration

## Abstract

Educational contexts play a critical role in identifying and responding to children impacted by trauma. However, with the multifaceted challenges experienced by teachers, this responsibility should not reside solely with them. This systematic scoping review examines the integration of cross-disciplinary collaboration in existing trauma-informed initiatives in early childhood and primary school settings. A systematic search of five online databases (ERIC, PsycINFO, Medline, CINAHL, and A+ Education) resulted in 28 articles that met the inclusion criteria. Characteristics, components, reported enablers and barriers, and outcomes evaluated of cross-disciplinary collaboration in the context of trauma-informed practice were explored. Findings suggest a limited understanding of cross-disciplinary collaboration as a specific approach to support trauma-impacted children in educational settings. Nevertheless, there is emerging evidence of its presence in trauma-informed initiatives, through forms including coaching, consultation, co-delivery of manualized curricula/interventions, and co-screening of students’ trauma backgrounds. Notably, co-screening of student trauma is observed only in primary schools, highlighting a gap to explore in early childhood education. Meanwhile, our knowledge of the effectiveness of this approach is limited, suggesting a need for further exploration using rigorous methodologies to build a robust evidence base. This will inform the development of more comprehensive and sustainable trauma-informed practices that effectively support trauma-impacted children in educational settings. Further, understanding of the enablers and barriers to cross-disciplinary collaboration at both professional and agency levels remains insufficient. This review underscores the nascent yet promising role of cross-disciplinary collaboration to support trauma-impacted children in Early Childhood Education and Care and primary school settings and suggests key areas for future exploration.

## Introduction

### Background

The prevalence of children experiencing adversity and trauma is increasingly recognized internationally (Mathews et al., 2023), with growing awareness on the critical role educational settings can play to prevent, identify, respond to, and support these children (Bartlett & Smith, 2019; Loomis, 2018; Sun, Tamblyn et al., 2024). As a result, a proliferation of programs has been developed to support the adoption of trauma-informed practices in educational settings, with positive impacts on children, teachers, and organizations (Sun, Blewitt et al., 2024). Looking at the components of these trauma-informed initiatives, recent systematic reviews of trauma-informed programs in early childhood education and care (ECEC) (Sun, Blewitt et al., 2024) and schools (Champine et al., 2019) suggest that the current efforts primarily focus on upskilling teachers. For example, programs such as Enhancing Trauma Awareness Program (Whitaker et al., 2019) emphasize equipping educators with knowledge, confidence, and strategies to use trauma-informed approach in their teaching practices. While such approaches are essential, there has been comparatively less exploration of how external resources (e.g., professionals specialized in trauma) can collaborate with schools and early childhood services to support the trauma-informed changes. Expanding this focus to explore cross-disciplinary collaboration could address broader challenges faced by educators in educational organizations.

### Challenges Experienced by Teachers

Teachers experience multiple challenges when working with trauma-impacted children. A thematic synthesis of qualitative studies that explored early childhood and primary teachers’ experiences of trauma-informed practice over the past decade suggests that (a) teachers are unprepared and experience challenges due to lack of preparation and supports; (b) working with trauma-impacted children without support can impact on their well-being; (c) teachers experience difficulties meeting the needs of all children; and (d) teachers are unsure about their professional roles and boundaries in response to trauma-exposed students (Sun, Tamblyn et al., 2024). It is widely acknowledged that teachers’ preparation, capability, and capacity to respond to students’ trauma are not adequate (Rodger et al., 2020; Rosenau, 2021). This is reflected in their limited knowledge, confidence, and skills in identifying trauma, responding to students who have experienced trauma, making referrals to external professionals when needed, and managing the impact of trauma on their own well-being (Sun, Tamblyn et al., 2024). Initiatives advocating for trauma-informed practices to be included in initial teacher education are increasingly evident (L’Estrange & Howard, 2022). While this is a promising step toward trauma-informed education, it is essential to understand the multiple barriers (i.e., lack of training, support, time, and resources) that teachers encounter, which can make it challenging for them to address all children’s needs on their own (Ekornes, 2015). Cross-disciplinary collaboration was identified as an area to explore from a systematic review and thematic synthesis of early childhood and primary school teachers’ experiences and needs in working with children experiencing trauma (Sun, Tamblyn et al., 2024). Emerging research perspectives argue that addressing the needs of trauma-impacted children requires the collective effort and expertise of various professionals, spanning education, health, and social care sectors (Loomis et al., 2023). Social care, in this context, refers to support services for children and families facing challenges and disadvantages, such as child protection, family support, and community-based services, often delivered by professionals such as social workers and case managers. Indeed, the responsibility for supporting trauma-affected children in educational settings should not reside with teachers alone. How cross-disciplinary collaboration can be enacted to support teachers in providing trauma-informed education and care to trauma-impacted children and benefit all children is thus crucial.

### Cross-Disciplinary Collaboration in Education

Cross-disciplinary collaboration is increasingly endorsed in early childhood policy. For example, the Early Years Strategy in Australia (Australian Government, 2024) emphasized the significance of having “collective efforts for a coordinated approach to children’s well-being and development” (p. 9). Similarly, the updated Act on ECEC in Finland (Ministry of Education and Culture, 2021) stated the need to promote interprofessional collaboration in early childhood education. However, the diverse terminologies around collaboration (e.g., multidisciplinary, interdisciplinary, transdisciplinary, interprofessional, interagency, and cross-sector) have been inconsistently interpreted and used. In this review, the term cross-disciplinary collaboration will be used as an overarching term. This will encompass various terms with different characteristics, denoting the collaboration of at least two professionals from different disciplines/sectors (e.g., early childhood and mental health services) working together to support children in educational context. While the implementation and operation of cross-disciplinarily collaboration have been insufficiently investigated in educational contexts, including ECEC and schools (Fukkink & Lalihatu, 2020), preliminary findings suggest several benefits, such as: (a) improved service provision; (b) reduced fragmentation of services along disciplinary lines and service duplication; (c) promotion of interventions within natural environments and routines; and (d) a shared understanding of child development as holistic and integrated (Hong & Reynolds-Keefer, 2013).

In health and social services sectors, cross-disciplinary collaboration has been growingly explored to break down siloes within these contexts. Similar recognition and efforts are not examined to the same extent in the educational context. The latest “the village” approach highlights the need to move past a traditional, siloed, profession-centered approach, to a more coordinated response from education, health, and social care when working with children (Reupert et al., 2022). However, existing barriers regarding the unclear characteristics of effective cross-disciplinary collaboration, and multiple challenges (e.g., conceptual challenges, policy differences; Bricker et al. 2022) may inhibit the adoption of this approach.

### The Need for Cross-Disciplinary Collaboration in Promoting Trauma-Informed Education

Trauma-informed care (TIC) is rooted in medicine, specifically in patient care back in the 1970s. Following the seminal adverse childhood experiences (ACEs) study (Felitti et al., 1998), the prevalence and multifaceted consequences of trauma gained a burgeoning awareness. This approach is then increasingly explored to be embedded in other settings, including education, with the guiding principles include (a) safety; (b) trustworthiness and transparency; (c) peer support; (d) collaboration and mutuality; (e) empowerment, voice and choice; and (f) cultural, historical, and gender issues (Substance Abuse and Mental Health Services Administration [SAMHSA], 2014). In educational contexts, researchers are increasingly emphasizing a multitiered approach to trauma-informed delivery, which provides varying levels of support based on children’s needs (Berger, 2019). Tier 1 focuses on universal strategies, such as upskilling the educational workforce and fostering trauma-informed environments that support all children, regardless of trauma exposure. Tier 2 offers targeted interventions for children at risk or exhibiting early signs of socio-emotional challenges, while Tier 3 provides individualized, intensive support for children with significant trauma-related concerns. Programs are considered multitiered when they incorporate more than one level of support (SAMHSA, 2014).

However, teachers, as professionals who work with students in these educational settings on a day-to-day basis, were not trained to adopt this trauma-informed approach to work with children affected by trauma (Lombardi-Davis, 2020; Sun, Tamblyn et al., 2024). Extensive research has documented the negative impact on educator well-being when supporting trauma-impacted children (e.g., burnout, secondary traumatic stress, and emotional exhaustion) (Berger & Nott, 2024), and the challenges they experience to adopt trauma-informed practices authentically (Brunzell et al., 2022; Pribitkin, 2020; Sun, Skouteris et al., 2024). These challenges encompass difficulties in recognizing trauma, managing behavioral issues, engaging with families, meeting the needs of both trauma-affected children and other children in the group, juggling multiple responsibilities, ensuring their own safety, facing a lack of trust, respect, and support from leadership, and becoming emotionally invested in children’s experiences leading to fatigue (Sun, Skouteris et al., 2024). A recent qualitative inquiry documented teachers’ expressed need for support from professionals having expertise in TIC (Sun, Skouteris et al., 2024). A cross-disciplinary collaboration approach, where teachers work with professionals with expertise in trauma-informed practices to support trauma-impacted children, may be helpful to build education professionals’ capacity, develop trauma-informed universal curriculum plan (e.g., environment set-up, intentional learning experiences), provide targeted support, and facilitate timely referrals as needed based on trauma screening and assessment.

Indeed, no single individual or organization can adequately meet the diverse needs presented by young children (Reeves et al., 2017). Collaboration with interdisciplinary professionals at either the internal (e.g., internal collaboration between school psychologist and teachers) and/or external (e.g., collaboration between teachers and external professionals) level can enable educational services to be equipped with the capacity to support students with complex needs (Boyland et al., 2019). Collaboration among interdisciplinary professionals, such as educators and health professionals, has demonstrated value by increasing pathways for sharing information, supporting the development of strategies that reflect interdisciplinary input, and enhancing the adaptability of service provision across teams when a child’s needs or circumstances change (Garvis et al., 2016). For children impacted by trauma, healing is not solely reliant on professional mental health treatment (O’Toole, 2022). Instead, communities can provide a relational nurturing environment, serving to protect the child against the negative impacts of trauma. Additionally, not all therapeutic strategies need to occur in traditional therapeutic settings; teachers can act as agents, supporting the co-planning and co-delivery of these strategies in natural contexts (Morton & Berardi, 2018). Meanwhile, exploring ways where teachers’ well-being can be supported, and professional capacity enhanced through such collaboration can enable them to better support all children. The universal accessibility and non-stigmatizing nature of educational settings make them an ideal environment for interdisciplinary professionals to collaborate in supporting children impacted by trauma. Thus, educational contexts have potential to be operationalized as public health “hubs,” anchoring cross-disciplinary collaboration, to support the integration of trauma-informed approaches, and provide holistic, integrated, and tailored support to children experiencing trauma. Such integration of trauma-informed practices into layered everyday teaching practice may have potential to benefit all children.

### Research Questions

It appears that cross-disciplinary collaboration may be beneficial to promote the adoption and inclusion of trauma-informed approaches in the educational context. To the best of our knowledge, this has not been well understood and explored systematically, nor has this been an explicit focus in trauma-informed initiatives. The aim of this scoping review was to explore if and how cross-disciplinary collaboration are embedded in current existing trauma-informed initiatives within early childhood and primary school settings, either internally (e.g., between teachers and school psychologists) or externally (e.g., between teachers and external social workers). The early childhood and primary school years are critical developmental periods when children are particularly sensitive to both adversity and support (Shonkoff & Phillips, 2000). During this time, foundational skills for emotional regulation, social interaction, and learning are established. For children who have experienced adversity and trauma, early childhood and primary schools, as universal services, can serve as safe and healing spaces. These educational settings offer unique opportunities to embed trauma-informed practices that foster resilience and well-being. However, despite their significance, there remains a notable research gap regarding cross-disciplinary approaches to addressing student trauma in these settings, which this review seeks to explore.

Specifically, the following research questions were addressed in this systematic scoping review: (a) What trauma-informed initiatives in early childhood and primary school settings incorporate a cross-disciplinary approach, and what specific terminologies did they use to describe collaboration? (b) What are the characteristics and components of cross-disciplinary collaboration within trauma-informed initiatives in early childhood and primary school settings? (c) What are the reported enablers and barriers to cross-disciplinary collaboration in supporting trauma-impacted children in early childhood and primary school contexts, if any? (d) What are the outcomes of cross-disciplinary collaboration in the context of trauma-informed practices known, if any?

## Method

A scoping review is a form of knowledge synthesis aimed at mapping the available evidence and identify characteristics addressing an exploratory research question (Pollock et al. 2023). Considering the nascent nature of including cross-disciplinary collaboration as part of the trauma-informed initiatives, the scoping review method was deemed appropriate to capture a wide range of literature (e.g., qualitative, descriptive and protocol papers for new interventions, quantitative, and program evaluation). Five steps were followed guided by the framework developed by Arksey and O’Malley’s (2005) and extended by Levac et al. (2010). These were: (a) identifying the research question(s); (b) identifying the relevant studies; (c) study selection; (d) data charting; and (e) collating, summarizing, and reporting results.

### Search Strategy

Three groups of key terms were combined to identify a comprehensive set of articles. The first concept was “trauma-informed,” the second concept was “cross-disciplinary collaboration,” and the third concept was “setting” (i.e., early childhood and primary) (see full search strategy in Table S1). In January 2024, a comprehensive search of five online databases, ERIC, PsycINFO, Medline, A+ Education, and CINAHL were conducted to identify relevant peer-reviewed articles in English published over the past decade (2013–2023). Since the publication of SAMHSA’s Concept of Trauma and Guidance for a Trauma-Informed Approach (2014), there has been a notable rise in the adoption of trauma-informed approaches and programs in educational settings. As such, the chosen search window (2013–2023) is well-suited to encompass relevant and current research. Manual searching of the reference list of included studies were also performed to minimize the possibilities of missing articles, with suitable articles included.

### Study Selection

Articles were included if the study described, discussed, or evaluated a trauma-informed initiative with cross-disciplinary collaboration component (e.g., programs, interventions, training, materials, and activities) in early childhood and primary school context; the collaboration could be internal (e.g., internal collaboration between school psychologists and teachers), or external (e.g., external collaboration between teachers and social workers). Study design was not restricted for the scoping purpose. Studies were excluded if: (a) the program does not focus on supporting early childhood and/or primary educational settings to be trauma-informed (e.g., articles need to explicitly define themselves as trauma-informed or the elements of the program are trauma-informed based on the six core principles); (b) the trauma-informed initiative does not include a cross-disciplinary collaborative component; (c) the collaborative trauma-informed initiative is outside of early childhood and primary school contexts (e.g., healthcare, social care, higher education); and (d) studies were published prior to 2013.

**Figure 1.**
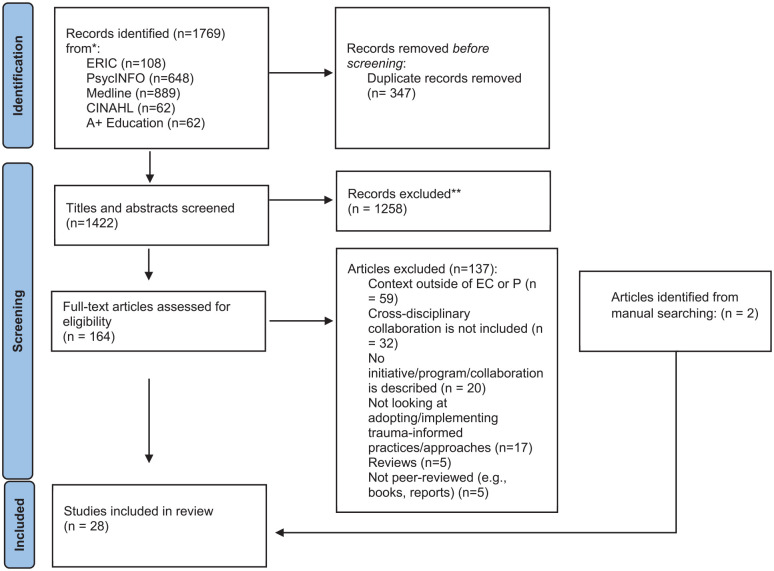
PRISMA chart.

A total of 1,769 records were yielded from the systematic search. Citations for all the articles were exported to the Covidence software and 347 duplicates removed. Two reviewers double screened 20% of titles and abstracts (*n* = 285) to refine the inclusion and exclusion criteria [YS & AT]. This process determined a conservative approach for the title and abstract screening stage due to the nuanced and often not evident aspect of cross-disciplinary collaboration in programs. Specifically, to ensure relevant studies were not missed, all trauma-informed initiatives for ECEC and primary settings were included for full-text review. Ultimately, a total of 1,422 titles and abstracts were screened against the inclusion and exclusion criteria, and the full texts of 164 articles were further examined for eligibility. Of these, 20% (*n* = 33) were double screened by two reviewers [YS & AT], achieving an interrater reliability of 88% and 100% consensus after discussion. One researcher then screened the remaining studies [YS]. The final set for this scoping review included 28 articles (see Figure 1).

### Data Charting and Synthesis

Data were charted from each study by the first author and cross checked by another author [CB]. Extracted data included study characteristics (author, year of publication, geographical location, study type, and aim), study type (theoretical framework, study design, methodology and methods, and participants characteristics), trauma-informed initiative description (program name, description, and components), cross-disciplinary collaboration description (characteristics, collaborative parties, and components), enablers and barriers (if any), evaluation outcomes (if any). These data were then synthesized in the results to address the proposed research questions. Specifically, program was categorized as Tier 1 if it seeks to provide universal support for all children, including the universal training for teachers regarding trauma-informed practices (Berger, 2019). Tier 2 was assigned if targeted support is provided for a small group of children, and Tier 3 was noted if intensive individualized intervention was provided for children with significant needs. Programs were noted as multitiered if more than one tier of support are included. Meanwhile, the action level of the cross-disciplinary collaboration was extracted. The professional level refers to the collaboration happens between interdisciplinary professionals (e.g., teachers and psychologists), while the agency level suggests the cross-disciplinary collaboration at the organizational level (e.g., early childhood organization and mental health agency).

## Results

### General Characteristics of the Included Articles

A total of 28 articles describing trauma-informed initiatives involving a cross-disciplinary collaboration component were included in this scoping review (Table 1). Of these, 11 studies focused on the ECEC context [reference number in Table 1: 1–11], and 17 targeted the primary school context [12–28]. All studies were published after 2015. The majority of the included studies were conducted in the United States (*n* = 24), with three in Australia, and one in Canada. In the present synthesis, 10 articles were descriptive only [2, 8, 9, 12, 15, 19, 20, 22–24], and 18 articles reported empirical study findings. Out of the empirical articles, eight adopted quantitative methods [5, 10, 11, 16, 17, 26–28], four applied qualitative approaches [1, 4, 13, 18], and six used mixed-methods design [3, 6, 7, 14, 21, 25].

**Table 1.** Characteristics of the Included Articles.

Article no.	Context	First author (year, country)	Study type	Program name	Program duration	Program description	Target population	Cross-disciplinary collaboration description	Terminologies
1	EC	Blewitt (2023, Australia)	Empirical—qualitative—realist evaluation	TraCS	Min 12 months	Trauma consultancy services delivered to ECEC tailored to the needs	Educators	TraCS consultants (trained in SW or psychology) and educators work together, with TraCS consultants provide support to educators as needed	Implicit
2		Natale (2023, US)	Descriptive—RCT protocol	JS + CS virtual toolkit	MHCs provide 24 weeks of teleconsultations for 4 hr weekly per center, divided between directors and teachers	MHCs to deliver a JS + CS virtual toolkit to childcare centers via a Kubi robot, to support psychosocial and safety needs	Childcare centers that serve low-income families from ethnic minority backgrounds (e.g., serve at least 60% Hispanic or 60% non-hispanic black families)	MHCs and educators work together through virtual consultation during covid-19, but more so MHC consultants provide support to educators	Implicit
3		Woods-Jaeger (2023, US)	Empirical—qualitative & quantitative	2Gen thrive	28 sessions	—Classroom theraplay: therapist-lead activities delivered in partnership with teachers. 28 sessions. —Dialectical behavior therapy skills training for parents	Low-income, minority families in the context of risk factors for toxic stress	An early head start program and a children’s hospital. Therapists work with teachers to deliver theraplay activities	Explicit Partnership, Collaboration
4		Douglass (2021, US)	Empirical—qualitative—multiple case study	TIC BSC	1–2 year period	Led by a certified “improvement advisor.” Planning phase—pre-work/launch—three learning sessions (with technical assistance and support: monthly call, metrics, website, site visits)	Five urban ECE programs that indicated they served children exposed to trauma	BSC coaches supported EC teams, focus more on organizational capacity building. Collaborative professional learning	Explicit Collaborative Interagency
5		Lipscomb (2021, US)	Empirical—RCT	Roots of resilience	Training: six modules Online coaching: six sessions	—Online course: six interactive, self-paced modules related to strengthening resilience of trauma-impacted children. —Video-based online coaching	Teachers who are supporting young children experiencing adversity	Teachers & coaches	Implicit
6		Lipscomb (2019, US)	Empirical—mixed method				ECT		Implicit
7		Woods-Jaeger (2018, US)	Empirical—mixed method	2Gen thrive	Classroom theraplay: 2× week for 7 weeks, 28 sessions total	See no. 3.	Infant and toddler classrooms at head start	Mental health therapist & teacher work together to deliver classroom theraplay to prevent toxic stress among children exposed to adversity by fostering nurturing relationships with their teachers	Explicit Collaborative Partnership
8		O’Malley (2017, US)	Descriptive	PRF	Continuous	PRF is designed to draw together the various disciplines to serve children and families using a multidisciplinary model for health and wellness care	Children growing up in poverty with risks of experiencing toxic stress	Collaboration between a ECEC and a children’s hospital that are close by. Nurse on site	Explicit Multidisciplinary Collaboration
9		Perry (2016, US)	Descriptive	PLAY	/	Teams a mental health professional with an EC provider in an ongoing problem-solving and capacity-building relationship	Teachers who support children experienced trauma	Project PLAY consultants & teachers Consultants directly work with teachers and directors to support trauma-impacted children from multiple aspects, including capacity building, environment set-up, routine, providing toolkit	Explicit Collaborative Team
10		Shamblin (2016, US)	Empirical—quantitative—single group pre-post	The twin efforts of the partnerships program for early childhood mental health and project launch	1 year	Embedded consultants in schools to increase capacity and positive supports for teachers + on-site mental health interventions delivered to children. A community-university-state partnership by pooling and disseminating critical resources, strengthening the skills, confidence and capacity of the early childhood education workforce	Children living in rural Appalachia experiencing poverty and adversity	Teachers & consultants School & mental health center	Explicit Collaboration Partnership
11		Holmes (2015, US)	Empirical—quantitative—single group pre-post	HSTS	1–2 years	—Training —Intensive individual trauma-focused intervention —Classroom consultation —Peer-based mentoring	Head start supporting children exposed to traumatic situations	HSTS therapists & teachers An early education/mental health cross-systems partnership	Implicit
12	P	Kotzin (2023, US)	Descriptive	The body’s story	Six sessions	A six-session manualized intervention, led by a SW as a trauma-informed supplement to SEL	All children	Led by a SW, supported by administrators, & teachers to deliver the intervention	Implicit
13		Somers (2022, US)	Empirical—qualitative—single-case study	Bayor Charter School	Continuous	A multidisciplinary team work across of teams: —Resilience team —Teacher PD —Expanding student support staff —Mental health services center on site —Equity team —Comprehensive school counseling advisory council	A high-need K–8 charter school	School counselors & social workers & leaders support teachers, but also share the load by working directly with students	Explicit Multidisciplinary Collaboration Partnership
14		Thierry (2022, US)	Empirical—case study with multiple data sources (both qual and quant)	/	3 years	Adult preparation or “readiness” for an elementary school’s adoption and implementation of a trauma informed, SEL universal curriculum	A school in a neighborhood with average income below poverty line Students: 69% African American, 17% Latinx or Hispanic, 7% White, and 6% two or more races; 16% English learner	School & mental health agency	Explicit Collaboration Partnership
15		Wisdom (2022, US)	Descriptive	TMI	Continuous	A community-based model that implements a comprehensive approach to ACEs and substance use prevention and mitigation by leveraging partnerships in public health and health care, public safety, and education	All students	Teachers & police officers work together to promote protective social norms through visits twice a year. Teachers work with SW to teach prosocial skills across lifespan through evidence-based program	Explicit Collaboration Across sector
16		Diggins (2021, Australia)	Empirical—quantitative—single group pre-post	ReLATE	Continuous	ReLATE promotes schoolwide trauma-specific interventions: —The community meeting —Safety plans —TCI, debriefing and reflective practice —Life space interview —Debriefing	Students who require high levels of adjustment in education	Teachers, education support, & school psychologists [internal] Psychologists provided training, assisted with debriefing & supervision	Implicit
17		Gilmer (2021, US)	Empirical—quantitative	/	Continuous	Regionally based community partnerships: community outreach, engagement, linkages to resources among community member, providing trauma-informed professional development for public school educators	Broad community members	MHCs & teachers Initiated and outreached by mental health agencies, supported/funded by governments. The focus is broad, with one target as schools and public-school educators	Explicit Partnership
18		Tremblay (2021, Canada)	Empirical—qualitative—community-based participatory	WRaP	Continuous—data were collected over the 4 years	Individualized, strength-based supports in schools to children and youth affected by FASD. Coaches were physically situated within their respective schools and also conducted outreach work in their communities	Students with FASD	School & Wrap Project coordinator & coach (training in social work or child and youth care)	Explicit Collaboration Partnership
19		Bryan (2020, US)	Descriptive	An equity-focused school—family—community partnership model	Continuous	Collaborative initiatives and relationships among school personnel, family members, and community members and representatives of community-based organizations. Partners collaborate in planning, coordinating, and implementing programs and activities at home, at school	Urban, low-income, and racially/ethnically diverse students	School counselor & other school staff (including teachers) & family [internal] Mainly described how school counselor can build equity-focused partnerships with families, but teachers also play a role in this. For example, school counselor does needs assessments with teachers	Explicit Collaboration Partnership
20		Clements (2020, US)	Descriptive	The Johnson City Tennessee System of Care	Continuous	A community-wide system of TIC enhancing collaboration and common language across sectors and organizations within sectors. A toolkit, offers rationale, step-by-step instructions, and tools (e.g., training materials, assessments instruments). A three-prolonged plan (begin with one champion, advocate, educate, collaborate) to streamline replication	Broad focus across healthcare, education, children’s services, the faith community, behavioral health providers, criminal justice, law enforcement, private businesses	The initial push came from inside a state government. Schools are part of the collaborative	Explicit Collaboration Partnership Across sector
21		Báez (2019, US)	Empirical—mixed method—explanatory sequential design	Wediko	Continuous Providers receive min 3-hr clinical supervision per week	A schoolwide multitiered trauma-informed approach to provide SEL programming, milieu support, and counseling to students, resources for families, and professional support to staff. Tier 1: schoolwide supports & SEL; Tier 2: group counseling & supports; Tier 3: individualized counseling & case management	All students	Social workers & mental health counselors & school staffs School & community-based organizations	Explicit Partnership
22		Von Der Embse (2019, US)	Descriptive	/	Training: 2 hr multimedia interactive workshops coaching: weekly	Multitiered mental health assessment to intervention model	All students in a large urban school district	Teachers & School Psychologists collaborate to upskill teachers in universal screening/assessment & effective classroom management practices	Implicit
23		Kataoka (2018, US)	Descriptive	/	Continuous	Three cases: 1: SMH and academic partners co-created and piloted an online progress monitoring and cloud-based care management tool that allows SMH to track students across tiered services and screen students electronically. 2: Whole school approach: creating a community health advocates schools, where TI principles woven into curriculum. 3: Harmony elementary school	All students	Case 1: teachers & academic clinician researchers & social workers. Schools facilitate cross-sector collaboration with child welfare and probation, community agencies, and mental health facilities. Case 2: collaboratively create a trauma-informed school, with teachers, principals, counselors, and support staffs	Explicit cross-sector collaboration
24		Morton (2018, US)	Descriptive	/	Continuous	Mental health professionals assist educators in developing trauma-informed competencies, through training, collaboration to implement, peer consultation & coaching, co-assessment	Educators and mental health professionals supporting children experienced trauma or chronic stress	Mental health professionals & educator partnerships	Explicit Multidisciplinary collaboration
25		Perry (2016, US)	Empirical—mixed method	NHTC	PD: 2 full days Care coordination: continuous Classroom workshop: 3 days	—PD —Care coordination —Clinical services: classroom workshops —Clinical services: cognitive behavioral intervention	Students impacted by trauma in schools	More so agency-level partnership, individual-level collaboration was not specifically described	Explicit Coordination Coalition
26		Iizuka (2015, Australia)	Empirical—quantitative—single group pre-post	Friends for life	PD: one full day Program: diverse tailored to the needs of participants and school curriculum	The PD + coaching model	Teachers in a school in a low socio-economic status area	Teachers & coaches	Implicit
27		Tabone (2020, US)	Empirical—pre-test post-test control group design	TIES	Three school years	—Teacher training —Classroom consultation —Family engagement and outreach	Students and teachers in 94 classrooms (i.e., pre-k, k, first grade) in rural West Virginia where high rate of parental substance use is present	Teachers & resource liaisons (i.e., licensed therapist)	Implicit
28		Rishel (2019, US)	Empirical—pilot—pre-test post-test control group design		Two school years		Students and teachers in 51 classrooms (i.e., pre-k, k, first grade) in West Virginia, a rural state overwhelmed by the opioid epidemic	Teachers & resource liaisons (i.e., licensed therapist)	Implicit

*Note*. TraCS = trauma consultancy service; ECEC = early childhood education and care; EC = early childhood; RCT = randomized clinical trial; MHCs = mental health consultants; TIC = trauma-informed care; BSC = breakthrough series collaborative; ECE = early childhood education; PRF = partnership for resilient families; TIC BSC = TIC the breakthrough series collaborative; 2Gen Thrive = two generations thrive; JS + CS = jump start+: COVID 19 support; PLAY = positive learning for Arkansas’ youngest; HSTS = head start trauma smart; TMI = the Martinsburg initiative; ReLATE = the rethinking learning and teaching environments; WRaP = the wellness, resiliency, and partnerships; Wediko = Wediko community school partnership; NHTC = New Haven trauma coalition; TIES = trauma-informed elementary schools; SW = social worker; ACE = adverse childhood experience; TCI = therapeutic crisis intervention; FASD = fetal alcohol spectrum disorder; PD = professional development; SEL = social emotional learning.

### Terminologies Used to Describe Collaboration

The collaborative terminologies used by the included articles are presented in Table 2. Seventeen articles explicitly used terms to denote collaboration [3, 4, 7–10, 13–15, 17–21, 23–25]. Among these, most studies used broad terms such as collaboration (*n* = 14) and partnership (*n* = 12). Coordination and coalition were used in one study each [25]. More specific terminologies such as interagency/cross-institutional (*n* = 2), cross-sector/multisector (*n* = 5), cross-disciplinary (*n* = 1), multidisciplinary (*n* = 4), and interdisciplinary (*n* = 1) were seen in less studies. It is notable that the terminologies around collaboration are complex, and each has a nuanced feature (Pfeiffer et al., 2019). Included studies rarely define and explain what they mean by collaboration, and how such cross-disciplinary collaboration was enacted in practice. Eleven articles did not explicitly use collaborative terms to denote collaboration, but the initiatives described having at least one interdisciplinary professional work with education staff in ECEC or primary schools to provide trauma-informed support to children [1, 2, 5, 6, 11, 12, 16, 22, 26–28].

**Table 2.** Collaboration Terminologies Used [3, 4, 7–10, 13–15, 17–21, 23–25] *n* = 17.

Terminology	Article used	Percentage
Coordination	25	5.88
Coalition	25	5.88
Collaborative/collaboration	3, 4, 7, 8, 10, 13–15, 18–20, 23–25	82.35
Partner/partnership	3, 8–11, 13, 14, 17–19, 21, 24	70.59
Interagency/cross-institutional	4, 7	11.76
Cross-sector/cross-system/ multisector	3, 11, 15, 20, 23	29.41
Cross-disciplinary	10	5.88
Multidisciplinary	13, 18, 20, 24	23.53
Interdisciplinary	10	5.88

### Cross-Disciplinary Collaboration in Trauma-Informed Initiatives

In response to the second research question, synthesized collaborative characteristics and components are presented in Table 3. Twelve programs were categorized as Tier 1, where the aim is to support teachers’ capacity building or provide universal support for all children attending the service. Among these, four focus on ECEC and eight target elementary school contexts. Only one initiative was categorized as Tier 2 (2Gen Thrive; [3, 7]), where the program was born in response to the high levels of trauma (i.e., toxic stress) experienced by children attending Head Start (Woods-Jaeger et al., 2018). Twelve initiatives were deemed as multitiered programs, with four target ECEC contexts, and eight support elementary schools. These programs are often not only multitiered but also multifaceted, with multiple components targeting different populations at various levels. For example, Wediko community school partnership is a schoolwide multitiered, trauma-informed approach to provide support tailored to the diverse needs of students, families, and staff (Báez et al., 2019). Wediko’s team of providers at each school includes social workers and mental health counselors to provide professional support for teachers, trauma-informed SEL programming, milieu support, counseling, and possible outreach for students, and resources for families. Schoolwide support and SEL are provided at tier 1, small group counseling and supports are provided at tier 2, and individualized counseling and case management are available at tier 3 (e.g., case consultation and referrals, individual counseling for students, home visits and family meetings, outreach phone calls). Cross-disciplinary collaboration is evident across all tiers in this program.

**Table 3.** Components, Action Levels, and Aims of the Collaboration.

Tier	Cross-disciplinary collaboration components										Action Level		Collaboration Aim(s)			Study
	C	Program	Collaborative parties	Coaching	Consultation	Manualized curriculum/intervention	Screening/assessment	Toolkit	Trauma service delivery	Other (specify)	Professional	Agency	Child	Teacher	Organization	
Tier 1	EC	JS + CS [2]	T & MHP		✓			✓			✓		✓	✓	✓	Natale
		Roots of resilience [5, 6]	T & coach	✓							✓		✓	✓		Lipscomb
		PRF [8]	Headstart & children’s hospital							Co-training, “huddles” between orgs, “sick bay” construction at childcare		✓	✓			O’Malley
		PLAY [9]	T & MHP		✓			✓			✓		✓	✓		Perry
	P	The body’s story [12]	T & SW			✓					✓		✓			Kotzin
		Thierry [14]	School & MH agency							“Readiness” building		✓	✓	✓	✓	Thierry
		ReLATE [16]	T & SP [internal]	✓	✓						✓		✓			Diggins
		Gilmer [17]	T & MHP S & MH agency							Community outreach, engagement, linkages to resources initiated by MH agencies	✓	✓		✓	✓	Gilmer
		Equity-focused school-family-community partnership [19]	T & SP [internal]				✓				✓		✓			Bryan
		The Johnson City Tennessee SoC [20]	Broad					✓		Enhancing collaboration and common language across sectors & orgs		✓			✓	Clements
		Morton [24]	T & MHP	✓	✓		✓			Implement concepts contextualized to the needs	✓			✓	✓	Morton
		FRIENDS for Life [26]	T & Coach	✓							✓		✓	✓		Lizuka
Tier 2	EC	2Gen Thrive [3, 7]	T & MHP Headstart & Children’s hospital	✓		✓					✓	✓	✓			Woods-Jaeger
Multitiered	EC	TraCS [1]	E & SW		✓						✓			✓		Blewitt
		TIC BSC [4]	T & MHP	✓							✓	✓		✓	✓	Douglass
		Partnerships program [10]	T & MHP								✓					Shamblin
		HSTS [11]	T & MHP Headstart & MH agency		✓						✓	✓	✓	✓		Holmes
	P	Bayor Charter School [13]	T & SW T & MHP T & SP [I + E] S & MH agency							Multidisciplinary teamwork across teams	✓	✓	✓	✓	✓	Somers
		TMI [15]	T& PO T & SW							Collaborative teaching	✓		✓			Wisdom
		WRaP [18]	T&SW	✓					✓		✓		✓		✓	Tremblay
		Wediko [21]	T & SW & MHP S & CO	✓	✓		✓				✓	✓	✓	✓		Báez
		Von Der Embse [22]	T & SP [Internal]	✓			✓				✓		✓	✓		Von der Embse
		Kataka [23]	T & SW T, L & SP S & child welfare & MH agency			✓	✓		✓		✓	✓	✓			Kataka
		NHTC [25]	S & gov & MH clinic				✓		✓	Care coordination		✓	✓	✓		Perry
		TIES [27, 28]	T & MHP	✓	✓		✓		✓	Resource liaison	✓		✓	✓		Rishel Tabone

*Note*. C = context; EC = early childhood; P = primary; JS+CS = jump start + Covid19 support; PRF = partnership for resilient families; PLAY = positive learning for Arkansas’ youngest; ReLATE = rethinking learning and teaching environments model; TraCS = trauma consultancy service; TIC BSC = trauma-informed care the breakthrough series collaborative; HSTS = head start trauma smart; TMI = the Martinsburg initiative; WRaP = the wellness, resiliency, and partnerships; Wediko = Wediko community school partnership; NHTC = new haven trauma coalition; T = teacher; MHP = mental health professionals; MH = mental health; SP = school psychologist; SW = social worker; PO = police officer; CO = community-based organization; gov = government.

### Characteristics of Cross-Disciplinary Collaboration

The majority of the cross-disciplinary collaboration captured in this review are operationalized at professional level only [1, 2, 5, 6, 9, 10, 12, 15, 16, 18, 19, 22, 24, 26–28], four occur at agency level only [8, 14, 20, 25], and eight seen at both professional and agency level [3, 4, 7, 11, 13, 17, 21, 23] (Table 4). Social workers and mental health professionals are the most common external professionals involved in the collaboration. Among mental health professionals, various sub-professions collaborate with educators to provide trauma-informed support for children. These include therapists [3, 7, 11, 27, 28], consultants [2, 7, 10, 17], counselors [21, 23], and school psychologists/counselors [16, 19, 22]. Specifically, all the cross-disciplinary collaborations within ECEC were external collaborations. Six studies described the collaboration between educators and mental health professionals [2, 3, 7, 9–11], two described the partnership between educators and social workers [1, 8], and one described how nurse can be on-site to work with educators in supporting children [8]. Three described the role of coach, without specific qualification mentioned, but mostly providing mental health support [4–6].

**Table 4.** Cross-Disciplinary Collaborative Parties and Action Levels.

Type of Collaboration	ECEC	Elementary School
Internal collaboration	/	School staffs (e.g., teachers, principals) & School psychologists/counselors: 13, 16, 19, 22
External collaboration	Professional level	
	Social workers: 1, 8	Social workers: 12, 13, 15, 18, 21, 23
	Mental health consultant: 2, 3, 10	Mental health consultant: 17
	Mental health therapist: 3, 7, 11	Mental health therapist: 27, 28
	Coach: 4–6	Mental health counselor: 21, 23
	Nurse: 8	Coach: 26
		Police officer: 15
	Agency level	
	Children’s hospital: 3, 7, 8	Mental health agency: 13, 14, 17, 23, 25
	Mental health agency: 4, 11	Child welfare: 21, 23
		Multisectors (broad): 20

*Note*. ECEC = early childhood education and care.

In elementary school contexts, it is notable that four studies presented an internal cross-disciplinary collaboration, where school psychologists/counselors would work collaboratively with teachers to provide trauma-informed practices to trauma-impacted children and all children [13, 16, 19, 22]. One initiative described both internal and external collaboration [13], where multidisciplinary collaboration across multiple teams was presented in a trauma-certified school (i.e., Bayor Charter School; Somers & Wheeler, 2022) to offer multiple systems of care for students. The remaining collaborations are all external between teachers and interdisciplinary professionals [*n* = 11, 12, 14, 15, 17, 18, 20, 21, 23–28]. Six studies demonstrated how social workers can work with school staff to provide trauma-informed services to students [12, 13, 15, 18, 21, 23], and eight described how mental health agencies or mental health professionals can partner with school staff [13, 14, 17, 21, 24, 25, 27, 28]. A police officer was involved in one aspect of one initiative, working with teachers to promote protective social norms by visiting schools twice a year [15]. One study described the role of coach, but no specific professional details were captured [26].

The common aims of the cross-disciplinary collaboration, as published in the studies, were to improve child-level outcomes (e.g., resilience, self-regulation, well-being, and psychosocial functioning; *n* = 19, 68%), followed by teacher-level objectives (e.g., capacity of using trauma-informed approaches, and well-being; *n* = 16, 57%), and organization-level aims (e.g., organization readiness, trauma-informed cultural change, policy and procedure, and community connectedness; *n* = 8, 29%).

### Components of Cross-Disciplinary Collaboration

Cross-disciplinary collaboration often takes many forms (see Table 3), with the level of collaboration differing across studies. The partnership between teachers and professionals with expertise in trauma are often multifaced, where these professionals play a critical role in supporting educators in developing trauma-informed competencies through training, coaching, consultation, co-assessment, and collaboration to implement trauma-informed strategies.

*Coaching* and *consultation* are common ways for professionals with relative expertise to support educators in: (a) capacity building (e.g., increasing awareness, knowledge, and strategies to provide trauma-informed education); (b) responding to the needs of trauma-impacted children collaboratively; and (c) promoting educators’ own self-care and well-being. Such forms of collaboration, especially consultations, often adopt a relational approach by acknowledging the challenges education professionals face when supporting trauma-affected students, and providing tailored support to them. For example, the TraCS program employs a flexible approach, including initial consultations, in-session and out-of-session consults, phone consultations, tailored training. These consultancy services are designed to align with the specific needs of the ECEC center, educators, and children (Blewitt et al., 2023). A similar initiative is project PLAY, where the formation of a collaborative relationship between an educator and a mental health consultant was the key. Consultants in this program work closely with educators to support children impacted by trauma through multiple aspects, such as educator capacity building, environment set-up, routine planning and establishment, and resourcing toolkits if needed (Perry & Conners-Burrow, 2016). In trauma-informed elementary schools (TIES) program, resource liaisons (i.e., experienced, licensed therapists) collaborate with classroom teachers to identify children exhibiting signs of trauma and create trauma-informed environment via classroom consultations (Tabone et al., 2020; Rishel et al., 2019).

Another form of cross-disciplinary collaboration is the *co-delivery of a manualized curriculum/intervention* by teachers and cross-disciplinary professionals [3, 7, 12, 23]. In 2Gen Thrive [3, 7], the music therapists work with teachers to deliver the music-focused theraplay activities to young children collaboratively, with teacher reported positive outcomes seen on child social engagement, child-initiated interactions, sense of trust and safety, self-regulation, and tolerance for new activity and people (Woods-Jaeger et al., 2018). *Screening/assessment* for trauma and gauging students’ need for additional support or referral is another form of cross-disciplinary collaboration, which is evident in seven programs captured [19, 21–25, 27, 28]. Interestingly, all these programs target elementary school contexts. Meanwhile, all initiatives including a cross-disciplinary collaboration to complete trauma screening are multitiered. Von der Embse et al. (2019) described a mental health assessment to intervention model, where an internal cross-disciplinary collaboration between teachers and school psychologists were presented. Teachers received training and coaching from school psychologists on universal screening and effective classroom management practices; such interprofessional collaboration is to integrate trauma-informed practices within the school setting. It is worth noting that screening/assessment is highlighted throughout the model to inform decision making to support students across all tiers (e.g., identify risks, evaluate responsiveness to intervention among students, and make data-based referral decisions if needed) (Von der Embse et al., 2019).

The other less commonly applied forms include *toolkit provision, application* [2, 9, 20], and *trauma service delivery* [18, 23, 25, 27, 28]. The provision of trauma-specific service is usually led by mental health professionals at a targeted (i.e., tier 2) or individualized (i.e., tier 3) scale. For example, in WRaP [18], individualized, strengths-based support for students with complex needs is the core (Tremblay et al., 2021). To do this, the coach is physically situated within the school to work with teachers in developing the plan and conduct needs-based outreach work in the community. Overall, regarding the nature of contact between educators and interdisciplinary professionals, seven articles described an embedded model. Of these, four focused on collaboration between education professionals and school psychologists, school counselors, and/or school social workers [13, 16, 19, 22], while three involving external social workers and/or mental health consultants (MHCs) embedded or co-located in educational settings [8, 10, 18]. Fourteen articles reported in-person, in-session collaboration without embedding [1, 3, 4, 7, 9, 11, 12, 15, 17, 22, 24, 26–28]. Virtual collaboration was described in five articles, including phone consultations [1, 4], video-based coaching [5, 6], and robot-assisted virtual consultations [2]. The remaining five articles did not specify the nature of contact [14, 20, 21, 23, 25].

Agency-level only collaboration is often broader in nature, with the aim not to directly support children, but more so to build organizational level trauma awareness, readiness, and capacity. Three initiatives included outreach from non-education organizations or systems (e.g., mental health agency) [14, 17, 20]. For example, the Johnson City Tennessee System of Care [20] aims to enhance collaboration and common language across sectors and organizations within sectors. To do this, they developed a toolkit, with instructions, resources, and implementation guide provided to maximize replication across sectors and organizations, with one recipient as schools and school staffs (Clements et al., 2020). However, no outcome was measured.

### Enablers and Barriers of Cross-Disciplinary Collaboration

All the included studies described a cross-disciplinary collaboration, either explicitly stated or implicitly implied. However, the “ingredients” of successful collaboration is less clear. Enablers and barriers experienced during the collaboration were not comprehensively reflected by authors. Nevertheless, articles with explicit collaboration focus were more likely to include a couple of lines discussing factors that facilitate or hinder the cross-disciplinary collaboration in the context of trauma-informed practices in educational settings. At professional level, data-based shared decision making has been suggested by five articles [13, 19, 21–23], followed by trusting relationship building between professionals [4, 18] and listening and communication [4, 13]. Learner mindset of collaborating professionals [8], clear common goals [19], information sharing [18], compromising [24], modeling [26], and progress celebration [19] were identified by one article each. At agency level, reported enablers include clear common goals of the collaboration [8, 15], trust [8, 17], information sharing between participating organizations [4], communication between organizational representatives [4, 8], leader commitment and buy-in [8, 15, 20], and consistent point of contact [20]. One barrier reported by O’Malley et al. (2017) suggested that with the shared common goals, the different organizational structures still get in the way, and a series complex negotiations and trust-building activities were needed.

### Outcomes of Cross-Disciplinary Collaboration

Eighteen out of the 28 included articles reported the empirical findings (see Table 5). Among these, 10 studies have outcomes reported directly linked to cross-disciplinary collaboration [1, 3, 7, 13, 14, 17, 18, 21, 27, 28]. Positive impacts on children, teachers, and schools were found. Overall, at the child level, positive impacts were reported by teachers on child behaviors [1, 21], child sense of safety and trust [3], and improved social engagement [3, 7]. However, Báez et al. (2019) found that students with higher reported levels of trauma reported more problem behaviors over the course of a school year in a trauma-informed community school partnership, despite receiving additional interventions. At the teacher level, improvements of teachers’ ability to use trauma-informed approaches [1, 13, 14, 18], increased confidence and well-being [1], and enhanced teacher–child interactions quality [7, 27, 28] were described. At the school level, improved school capacity [17], climate [14], and strengthened trauma-informed policies and procedures were reported [14] by school leaders who participated in the partnership. It is notable though that the majority (75%) of these outcomes evaluated were based on qualitative approaches [1, 3, 7, 13, 14, 18], with the rest used quantitative approaches ([17]; 12.5%) and mixed-method approach ([21]; 12.5%). Diversity characteristics, including ethnicity, socioeconomic status, and geographic location, were inconsistently reported in the reviewed studies, with only 55.5% (*n* = 10) providing such data. This lack of detailed demographic reporting limits the generalizability of the findings to broader populations.

**Table 5.** Outcomes Reported.

Article no.	Context	First author (year, country)	Study design	Measures/methods	Participant characteristics	Key outcomes reported	Collaboration measured specifically
1	EC	Blewitt (2023, Australia)	q—Realist evaluation	Interview	Thirteen TraCS consultants	Qualitative data generated eight program theories that capture how TraCS consultants support educators to develop a trauma-informed lens and practice. Each theory included the contextual factor, mechanism (resources + change in reasoning), and outcome. These theories highlighted how the consultants can work with educators to support both children and their own well-being. Outcomes at child level included (a) positive impact on child as expressed through their behavior. Outcomes at educator level included (a) increased awareness and understanding of behavior as an expression of a child’s experience, (b) increased educator confidence, (c) increased awareness and understanding of behavior as an expression of a child’s experience, (d) educators apply and integrate a trauma-lens to their work, (e) increased educator resilience and self-regulation, (f) improved educator well-being.	Y
3		Woods-Jaeger (2023, US)	M	Interview; pilot studies as per study no.7	Six therapists (66.7% female, 66.7% white) (EHS/HS clinical staff members, usually licensed clinical social workers or music therapists) 20 teachers (95% female, 45% white, 45% black)	Child level: —Teacher ratings of child outcomes in the classroom theraplay intervention suggested benefits in a range of domains, including: social engagement, child-initiated interactions with caregivers, sense of trust and safety, self-regulation, tolerance for new activities and people, and sense of attachment between child and caregiver. Parent level: —Significant reductions in depressive symptoms, parental distress, and difficulties with emotion regulation, with the overwhelming majority of parents reporting high satisfaction with the intervention and that it increased their confidence in their parenting skills	Y (Classroom theraplay component specifically)
4		Douglass (2021, US)	*q*—Multiple case study	Meeting observations; interviews; documents; BSC improvement tracking forms, monthly metrics, intranet posts	ECE Program Team Members (mid-point and post BSC); BSC Staff and Coaches (post-BSC)	Educator level: —expanded understanding and awareness of what it means to be trauma informed. —greater empathy for children, families, and staff. —increased confidence, empowerment, and teacher leadership. Organization level: —positive workplace relationships and shared leadership. —collaborative learning and use of data. —improved interagency collaboration.	N
5		Lipscomb (2021, US)	Q—RCT	—Teacher–child interactions: Classroom Assessment Scoring System (CLASS; Pianta, La Paro et al., 2008) —Children’s engagement: the Individualized Classroom Assessment Scoring System (inCLASS; Downer et al., 2010) —Children’s school readiness: Self-regulation: HTKS-R Emergent literacy & math skills: the Letter-Word Identification subtest of the Woodcock Johnson Tests of Achievement and their early math skills (ability to analyze and solve practical math problems) with the Applied Problems subtest of the Woodcock Johnson Tests of Achievement (Woodcock et al., 2001).	Twenty three teachers: 8.7% Latino or Hispanic and 95.7% White (91.3% White only). 61 children: 1.2% Native American, 4.9% Asian/Pacific Islander, 4.9% African American, 3.3% Latino, 91.8% White; 85% were White only.	Child level: —reductions in (observed) negative engagement and increases in direct assessments of math skills Teacher level: Significant increase on (observed) emotionally supportive teacher–child interactions.	N
6		Lipscomb (2019, US)	M	Feasibility: —Course (overall rate of completion of the course and time it took to complete each module reported by teachers) —Coaching (rated four items after each coaching session) Learning and application: —Couse: self-reports + quizzes (10 quizzes with 7–12 questions each, 89 total questions) + Qualitative data from discussion boards and workbooks —Coaching: self-reports + coach-reports + observations	Seventeen teachers: 94% female; 76% white	Preliminary evidence on teachers’ strengthened knowledge and application of trauma-informed practices to identify and respond to children’s needs.	N
7		Woods-Jaeger (2018, US)	M	—Therapist and teacher attitudes: A self-developed self-report instrument (a five-point Likert scale for questions about the benefits of theraplay) + therapist interview (semistructured) + teachers' brief feedback on their experience and recorded by the researcher with pen and paper + CLASS	Eighty six children predominately Black (79.1%) 20 teachers: 45%White, 45% Black 25 parents: 80% Black, 92% female	Child level: —Teachers reported (q) improvements in children’s expressiveness (from flat to expressive), increased interaction with caregivers and peers, and spontaneous singing of and initiation of activities, which demonstrated enjoyment and growing ability to initiate connections with caregivers and peers. Teacher level: —Rating scores demonstrated improvements across caregiving dimensions from baseline to post-intervention, including effective teacher–child interactions (highest category). Parent level: —Significant decrease of depressive symptoms and distress of parents	Y (Classroom theraplay component specifically)
10		Shamblin (2016, US)	*Q*—Single group pre-post	—Teacher confidence and competence: The teacher opinion scale (TOS;Geller & Lynch, 1999) —Quality of preschool environment: The preschool mental health climate scale (PMHCS; Gilliam, 2008) —Functional assessment of children: the Deverux Early Childhood Assessment (DECA; LeBuffe & Naglieri, 1999) —Teacher satisfaction and relationship with the consultant: the Georgetown University ECMHC satisfaction survey	Eleven teachers	Child level: —Significant higher resilience scores compared to control groups. Teacher level: —Significant pre-improvement/post-improvement in teacher confidence and hopefulness in positively impacting challenging child behaviors. Classroom level: —Significant reduction in the negative attributes of the preschool learning environment.	N
11		Holmes (2015, US)	*Q*—Single group pre-post	—The ability to pay attention: teacher report form of the Achenbach —Externalizing behavior and oppositional defiance: teacher report form of the Achenbach —Externalizing problems and attention/hyperactivity: CBCL parent report —internalizing behavior: CBCL parent report	Eighty one children: 31–76months, *M* = 4.25 years; 39% African-American, 15% non-Latino White, 8% Latino/ Latina, 3% other, data unavailable for about one-third (35%); 36% female	Children level: —Significant improvement in attention, externalizing behavior, oppositional defiance based on teacher report. —Parents reported significant improvements in externalizing problems and attention/hyperactivity, and internalizing behaviors.	N
13	P	Somers (2022, US)	*q*—single case study	—Semistructured interviews with school staff —EZ analyze time tracker (http://www.ezanalyze.com/), Power-School discipline logs and reports, advisory council minutes, staff professional learning records, web-based staff surveys, human resources records, resilience coach and behavior coach records, School Mental Health Quality Assessment data (National Centre for School Mental Health, 2019), SELWeb data (xSEL Labs, 2021), and counts of participants in various activities (e.g., SEL and counseling interventions).	School info: 56% children below poverty line; 14% African-American students, 29% Hispanic students, 11% multiracial students, and 46% White students; special education population 15.9%	Classroom level: —Utilization of social/emotional learning and trauma-informed lessons in the classroom. School level: —Reduced non-counseling duties for school counselors and providing 80% direct and indirect services to students; —Reduced school counselor-to-student ratio; —Restructured student discipline policies through the implementation of school-wide TIC practices.	Y (Case study of a school with multidisciplinary collaboration across teams)
14		Thierry (2022, US)	*M*	Case study	Twenty four teachers (pre-k to 5th-grade): 84.6% White, 15.4% African American; 96.2% female	Teacher level: —Strengthened SEL expertise. School level: —Improved positive school climate. E.g., revised school mission and vision to emphasize the importance of a safe environment, positive self-awareness, and empathy for others, that are displayed for parents and visitors. —Alignment of disciplinary policies and procedures.	Y (Case study of a school-mental health partnership)
16		Diggins (2021, Australia)	*Q*—single group pre-post	Positive & negative attributes of students: SDQ PTSD symptoms: PCL-PR	Eighteen students: 89% male; 22% Aboriginal or Torres Strait Islander; Some students had mild symptoms of trauma; three students exceeded the clinical cut-off	Child level: —Significant reductions were found for parent-reported conduct problems, peer problems and total difficulties. —New students to the school demonstrated more positive adjustment. —Existing students demonstrated nonsignificant positive adjustment. —A reliable change was found for Global Impact, which indicated the benefits that occurred from the intervention generalized into homelife, friendships, learning and leisure activities.	N (but discussed the role psychologist can play as consultants when implementing trauma-informed practice models in schools).
17		Gilmer (2021, US)	*Q*	—Trauma-informed trainings among partnership members: the Trauma-Informed Organizational Toolkit. —Outreach, engagement and linkages were documented using Event and Linkage Trackers. —Changes in stress tolerance capacities: the 10-item Conner-Davidson Resilience Scale; the 28-item Coping Orientation to Problems; and the pictorial Inclusion of Community in Self Scale.	Three hundred and thirty two partnership members	Organization level: —Community-based partnerships demonstrated effective capacity-building strategies. —Significant improvements in external help-seeking (use of emotional and instrumental supports) and perception of community connectedness.	Y
18		Tremblay (2021, Canada)	*q*—Participatory	Interview	WRaP coaches; four school principals	School level: —The WRaP project demonstrated significant growth and positive outcomes in terms of building school capacity to support students with complex needs including Fetal Alcohol Spectrum Disorder (FASD).	Y (Coach embedded in schools using an integrated, relational approach).
21		Báez (2019, US)	M	—Social skills & problem behaviors: SSIS-RS —10 ACEs: ACE questionnaire —Student interviews	Five hundred students evaluated Nine students interviewed: four male, five female	Child level: —Students reported lower social skills and higher problem behaviors as the level of reported traumatic experience increased. —Students with higher reported levels of trauma reported more problem behaviors over the course of a school year, in spite of receiving additional interventions.	Y (Trauma-informed community school partnerships).
25		Perry (2016, US)	M	—PD satisfaction survey —Student satisfaction survey —Trauma exposure and symptoms screener: the UCLA PTSD Index for DSM-IV adolescence version (Pynoos, Rodriguez, Steinberg, Stuber, & Frederick, 1998)	Pilot school students: 82% Black/African American, 13% Hispanic/Latino, and 5% White; predominantly low-income population; 76% of students are eligible for Free Lunch while another 5% are eligible for Reduced Lunch PD participants: 32 teachers and/or administrators Care coordination participants: 19 families with difficulties related to basic needs such as poverty, chronic unemployment, unstable housing, food scarcity, and/or limited community support Clinical service participants: two 5th grade and two 6th grade classrooms (*n* − 77)	Child level: —Learn skills in how to cope with current symptoms and how to respond to future stress. Teacher level: —School staff and/or community members in learning about trauma sensitive practices; —Identifying students in need of trauma-informed support; —Implementing systems to provide trauma-informed services to students.	N
26		Iizuka (2015, Australia)	*Q*—single group pre-post	Student assessments: —SDQ: emotional difficulties, conduct problems, hyperactivity & inattention, peer difficulties, pro-social behavior. —Spence Children's anxiety scale (SCAS): anxiety symptoms —FRIENDS Social Acceptability Measure: Acceptability of the program Teacher assessments: —Resilience scale: adults resilience Depression, Stress, and Anxiety—DASS-21 (the DASS-21): emotional states of depression, anxiety and stress —Teaching Satisfaction Scale—TSS: The TSS: teaching satisfaction of teachers —FRIENDS Social Acceptability Measure: Acceptability of the program	Sixty nine students: 26 boys, 43 girls 23 teachers: 18 females, 5 males	Child level: —Students who were at risk showed significant decrease in their anxiety levels. Teacher level: —Teacher’ s demonstrated significant improvements on their emotional resilience.	N
27		Tabone (2020, US)	*Q*—control group pre-post	CLASS	Seventy four pre-K, K, and 1st-Gradeclassrooms in 11 schools	Teacher level: —IG classrooms demonstrated a significant increase in emotional support (*p* = .00), classroom organization (*p* = .00) from baseline to end of school year improvements in promoting trauma-sensitive classroom environments from baseline to post intervention, with no such improvements in CG. —Compared to CG, IG classrooms showed significantly higher scores regarding emotional support (*F* = 13.07, *p* = .00), classroom organization (*F* = 5.52, *p* = .02), instructional support (*F* = 4.21, *p* = .04) after adjusting for baseline scores.	Y
28		Rishel (2019, US)	*Q*—control group pre-post	CLASS	Fifty one classrooms (including pre-K) in 11 schools	Teacher level: —IG classrooms demonstrated a significant increase from baseline to the end of school year in emotional support (*p* = .00), classroom organization (*p* = .00), whereas no such improvements were observed in CG classrooms. —Compared to CG classrooms, statistically significant difference was found in emotional support (*p* = .00) and classroom organization (*p* = .00), the difference in instructional support was not significant.	Y

*Note. Q* = quantitative; *q* = qualitative; *M* = mixed methods; *Y* = yes; *N* = no.

## Discussion

Education contexts are critical points for prevention and early intervention for trauma-impacted children, yet the responsibility should not reside with educators solely. Previous qualitative research with teachers identified the need for cross-disciplinary professionals with expertise in trauma to work with education professionals collaboratively in supporting trauma-impacted children through integrating trauma-informed approaches in educational settings (Sun, Skouteris et al., 2024). However, our understanding of this cross-disciplinary collaboration approach in the context of trauma-informed education is limited. This review explored if and how cross-disciplinary collaboration is incorporated in trauma-informed initiatives in early childhood and primary school settings, either explicitly or implicitly. Characteristics (i.e., collaboration-related terminologies, participating parties, action levels, and aims of the collaborations), components, reported enablers and barriers, and outcomes of cross-disciplinary collaboration were examined and synthesized.

It is promising that this review has uncovered existing trauma-informed initiatives that included collaboration between educators and interdisciplinary professionals across both ECEC and primary school settings as an important first step. The most common ways include coaching, consultation, manualized curriculum/intervention co-delivery, co-assessment, toolkit implementation, and trauma service delivery. Among the 28 included studies, eleven articles described cross-disciplinary collaboration implicitly. For articles that explicitly described collaborative efforts, the terminologies adopted were mostly broad in nature (e.g., collaboration, partnership), with less specific collaboration model described (e.g., transdisciplinary collaboration, interprofessional collaboration). As a result, the components and processes of such collaboration often lack detailed description and clarity. This might imply that the cross-disciplinary collaboration approach has not been well-understood and explored by program developers for trauma-informed initiatives in education. Preliminary positive outcomes of cross-disciplinary collaboration in trauma-informed initiatives have been documented for students (e.g., improved student sense of safety, positive student behaviors), teachers (e.g., professional capacity to provide trauma-informed education, improved teacher well-being), and organizations (e.g., readiness to be trauma-informed, policy and procedure improvement) through various ways. However, these findings were largely based on qualitative methods with small sample sizes, and many included studies have not evaluated the outcomes of this collaboration yet. Cross-disciplinary collaboration, as desired by teachers (Sun, Tamblyn et al., 2024), could be a promising implementation strategy to support the incorporation of trauma-informed practices in educational settings, with the potential to benefit students, teachers, and organizations. However, our understanding of the effectiveness of this approach, the context in which it works, and the mechanism behind its success is still limited. It is necessary to continuously explore this approach using rigorous methodologies as the next step to build a robust evidence base.

In the context of global policies advocating for the integration of health, education, and social care (Australian Government, 2024), and growing recognition among trauma researchers of the need for collective efforts (Loomis et al., 2023), the exploration of enablers and barriers to cross-disciplinary collaboration for promoting trauma-informed practices is still at its infancy. This includes understanding the determinants at both professional and agency level, as enabling organizations can facilitate smoother cross-disciplinary collaboration among professionals. There is a clear need to investigate how cross-disciplinary collaboration can be effectively enacted in educational settings to support trauma-impacted children, and embed trauma-informed practices into normalized daily practices. Few studies have examined the cost-effectiveness, feasibility, and other aspects of implementing such collaboration in early childhood and primary school settings. While virtual contact methods, such as phone consultations and video-based coaching, have been discussed as more scalable and cost-effective compared to in-person models (Lipscomb et al., 2019), further research is needed to understand how different modes of collaboration affect feasibility and sustainability.

In particular, in the collaboration captured in this review, teachers were often the one receiving support from interdisciplinary professionals in educational settings, potentially placing them in a lower side of the power dynamics. Previous studies have raised the concern that the historical undervaluation of education professionals may lead to teachers’ contributions being less valued in a cross-disciplinary team (Wong et al., 2012). Albeit the fact that often times teachers are not trained in TIC, they have extensive knowledge and expertise in child development and teaching pedagogy. Growing literature discussed the alignment between trauma-informed practices and high-quality teaching practice (Sun, Skouteris et al., 2024). For example, nurturing teacher–child interactions, consistent and predictable routines (Brunzell et al., 2016), and fostering social and emotional skills are at the core of trauma-informed practices (Osher et al., 2021). Meanwhile, as someone who spent extensive time with children, teachers are in a position to understand children’s individual interests, preferences, and capacities. These are important in planning targeted support for students. In a framework proposed by Bricker et al. (2022), collaborative practices encompass (a) communicating, (b) sharing, (c) joint planning, (d) contributing, (e) compromising, (f) modeling, and (g) acknowledging, highlighting the two-way collaboration where all members’ expertise is valued and incorporated. Building on this, our review adds the importance of trusting relationship building between collaborating professionals, and data-based shared decision-making. These elements may be helpful to consider for future trauma-informed initiatives when planning for cross-disciplinary collaboration. It is crucial to continuously advocate for and empower teachers’ professional expertise and status to achieve more authentic cross-disciplinary collaboration.

The emphasis on the relational approach is a recurring theme in the reviewed articles, addressing both interprofessional relationships and the establishment of a trauma-informed environment for children. Trauma-informed approaches are inherently strengths-based, grounded in an understanding of and responsiveness to the impact of trauma through the cultivation of physical, psychological, and emotional safety (Harris & Fallot, 2001; SAMHSA, 2014). Similarly, a relational pedagogy values the development of safe and trusting relationships characterized by support, non-judgment, compassion, and respect, thereby enhancing child outcomes (Bingham & Sidorkin, 2004; Koloroutis, 2004). These two approaches overlap in that safety and relationship are held in the center of the practice. Trusting relationship between professionals is one key feature of successful interprofessional collaboration, enabling smoother communication, joint planning, and co-delivery (Pullon, 2008). Meanwhile, adopting a relational approach to support trauma-impacted children shows positive effects on children’s emotional regulation, confidence and resilience (Wall, 2020). Thus, the relational approach for both cross-disciplinary collaboration and trauma-informed support for children is highly valued and should be continuously explored and advocated.

Several learnings can be drawn from cross-disciplinary collaboration to promote trauma-informed practices in primary school contexts to ECEC. To start with, collaboration to equip teachers with knowledge skills in trauma assessment/screening, and provide co-screening is seen in primary schools only. This highlights a clear gap of lacking focus on trauma screening and assessment for young children in ECEC. Having the capacity to screen or co-screen against trauma is an essential skill for teachers to identify students who may need additional support. This screening can take various forms, such as child observation, standardized assessment, and caregiver interviews (Eklund et al., 2018). Continuous exploration of how educators in ECEC can be supported to screen trauma is needed. Additionally, apart from collaboration with external multidisciplinary professionals, primary schools also have internal multidisciplinary collaboration which can provide more holistic perspectives and support for students. The valuable role of school psychologists in integrating trauma-informed practices into school context has been increasing recognized, where their professional expertise and relationship with both students and teachers place them in an ideal position to drive trauma-informed change (Howell et al., 2019; Ormiston et al., 2020). However, ECEC often lack the resources to employ a mental health professional onsite. For centers supporting high vulnerability populations, the co-location of a psychologist might be a viable option to explore. Finally, trauma-informed programs in primary schools are often multitiered and multilayered, highlighting an opportunity to promote more holistic and integrated trauma-informed initiatives within ECEC. However, the contextual and structural differences between primary school and ECEC need to be considered when interpreting these results for future cross-disciplinary collaboration planning in the context of trauma-informed education.

The studies included in this review primarily focused on communities where adversity and trauma are more prevalent, such as rural areas or low-income populations, with limited representation of urban settings. While this focus is important, trauma does not discriminate and can affect individuals regardless of age, gender, socioeconomic status, ethnicity, or geographic location (SAMHSA, 2014). It is true that prevalence is challenging to determine (Saunders & Adams, 2014), particularly in educational settings where awareness of trauma is still developing. Given that trauma-informed approaches have the potential to benefit all children and educators, future cross-disciplinary efforts should be trailed in diverse communities, including both urban and rural areas and across socioeconomic spectrums, to establish a more robust evidence base. It also remains unclear whether cross-disciplinary collaboration among professionals is enacted equitably. For example, professionals at different career stages (e.g., graduate teachers vs. experienced teachers) or those from culturally and linguistically diverse backgrounds may engage differently in such collaborations. Intentional efforts to ensure diversity, inclusion, and equity are essential.

## Limitations

This review systematically explored a nuanced aspect—cross-disciplinary collaboration, within trauma-informed initiatives. However, the inclusion criteria, which focused on this collaboration component, may have led to the omission of relevant studies that did not explicitly describe it in their titles and abstracts. A key strength of this review is the researchers’ extensive subject-matter expertise, allowing them to identify this potential limitation during the early pilot title and abstract screening stage. Hence, to maximize the replicability of this systematic scoping review, a conservative approach was adopted to include any trauma-informed initiatives for ECEC and primary school settings at the title and abstract screening stage. Full texts were then thoroughly reviewed to ensure that descriptions of cross-disciplinary collaboration, whether explicit or implicit, were captured for synthesis and analysis. This approach was validated as 11 out of 28 included articles implicitly described the cross-disciplinary collaboration within trauma-informed initiatives, confirming the merit of this conservative screening strategy. Meanwhile, this review captures the approaches for both ECEC and primary schools. While several applicable learnings can be drawn from primary school context to ECEC, the differences between these two settings are also evident. Careful considerations on the contexts are needed when interpreting the results of this scoping review.

## Conclusion

This systematic scoping review underscores the promising yet underexplored role of cross-disciplinary collaboration in advancing trauma-informed practices within early childhood and primary school settings. While evidence of its implementation is emerging, particularly in primary education, substantial gaps remain in both the conceptual understanding and practical application of these cross-disciplinary approaches to support trauma-impacted children and the educators who work with them. The effectiveness of cross-disciplinary collaborations in improving outcomes for both children and professionals remains under-evaluated and requires rigorous investigation. To address this, future research must employ robust methodologies to build a strong evidence base. Findings also highlight the need to elucidate the mechanisms, enablers, and barriers to effective collaboration across disciplines and sectors. Addressing these gaps will deepen our understanding of how cross-disciplinary collaboration approaches can facilitate the adoption and sustainability of trauma-informed practices in early childhood and primary school settings.

**Table:** Key Findings of the Review.

• Cross-disciplinary collaboration is increasingly seen in trauma-informed initiatives, through forms that include coaching, consultation, co-delivery of manualized curriculum/intervention, co-screening, and collaboration to implement trauma-informed strategies. • Co-screening is observed only in primary schools, highlighting a gap for ECEC. • Researchers often use broad terminologies instead of specific ones, with 35% of articles describing the collaboration implicitly, implying a lack of understanding of cross-disciplinary collaboration as a specific form to support trauma-impacted children in educational settings. • Preliminary positive outcomes of cross-disciplinary collaboration in the context of trauma-informed practices have been documented for students, teachers, and organizations. However, these findings were largely based on qualitative methods with small sample sizes. Our understanding of the effectiveness of this approach, the contexts in which it works, and the mechanism behind its success is still in its infancy. Therefore, it is necessary to continuously explore this approach using rigorous methodologies as the next step to build a robust evidence base. • There is insufficient understanding of the enablers and barriers to cross-disciplinary collaboration at both professional and agency level to support trauma-impacted children in educational settings, necessitating further investigation.

**Table:** Implications for Research, Practice, and Policy.

Research implications
• Cross-disciplinary collaboration was desired by teachers to support children impacted by trauma. However, our understanding of the effectiveness of this approach, the contexts in which it works, and the mechanism behind its success is still in its infancy. There is a crucial need to continuously build the evidence base for cross-disciplinary collaboration as an approach to embed trauma-informed practices in educational settings. • Understanding the enablers and barriers at both professional and agency levels for effective cross-disciplinary collaboration to embed trauma-informed approaches within educational settings is required. • Exploring ways to promote trauma screening and co-screening in ECEC is needed.
Practice and policy implications
• Teachers’ professional expertise and status should be continuously advocated toward a more authentic cross-disciplinary collaboration. • Prioritizing a relational approach for both cross-disciplinary collaboration and trauma-informed support for children is valued. • Continuous exploration of how educators can be supported to screen trauma in ECEC is needed.

## Supplemental Material

sj-docx-1-tva-10.1177_15248380251325217 – Supplemental material for Cross-Disciplinary Collaboration to Promote Trauma-Informed Practices in Early Childhood and Primary Education Supplemental material, sj-docx-1-tva-10.1177_15248380251325217 for Cross-Disciplinary Collaboration to Promote Trauma-Informed Practices in Early Childhood and Primary Education by Yihan Sun, Helen Skouteris, Andrea Tamblyn, Emily Berger and Claire Blewitt in Trauma, Violence, & Abuse
