# Supplementary material for: Cross-Disciplinary Collaboration to Promote Trauma-Informed Practices in Early Childhood and Primary Education
Source: Trauma Violence Abuse. 2025 Mar 27;27(3):775–95. doi: 10.1177/15248380251325217 (PMC13287515; doi:10.1177/15248380251325217)
Supplement: sj-docx-1-tva-10.1177_15248380251325217 – Supplemental material for Cross-Disciplinary Collaboration to Promote Trauma-Informed Practices in Early Childhood and Primary Education [file sj-docx-1-tva-10.1177_15248380251325217.docx]

**Table S1**

*Search Terms*

| **Concept** | **Trauma-informed** | **Cross-disciplinary Collaboration** | **Context (early childhood + primary school)** |
| --- | --- | --- | --- |
| **Terms** | trauma-informed OR  trauma-sensitive OR  trauma-aware OR  trauma-responsive OR  “trauma service” OR  trauma OR  “adverse childhood experience” OR  ACEs OR  adversity OR  resilience OR  safety-promoting OR  healing | cross?sector OR multi?sector OR multi?disciplinary OR inter?disciplinary OR interprofessional OR cross?disciplinary OR trans?disciplinary OR “key worker model” OR consult* OR collaborat* OR partnership OR teamwork OR cooperat* OR partner OR coordination OR coalition OR coaching OR colocation | School OR  “elementary school” OR  “primary school” OR  Education OR  learn* OR  pedagog* OR  “early education” OR  pre?school OR  child?care OR  kinder* OR  kindergarten OR  pre?kinder OR  pre?k OR  day?care OR  Head?Start OR  (“early childhood” adj2 education) OR  “early childhood” OR  nursery |

*Note: Different terms were used in each database for proximity searches. E.g., ADJ was used in Ovid Medline, psycinfo, and Embase; NEAR was used in ERIC; ~n was used in A+ Education; w/n was used in Scopus.*
